# Hemorrhage from the Bowels

**Published:** 1878-01

**Authors:** E. F. Starr

**Affiliations:** Nacoochee, Ga.


### HEMORRHAGE FROM THE BOWELS.

By E. F. STARR, M.D., Nacoochee, Ga.

During the course of my experience I have met with four cases of frightful hemorrhage from the bowels. Three of these occurred during the course of quasi typhoid fever, the other was without any such complication, and all of which recovered. Two were males and two females. Of the three fever cases, two were females. None of them had previously shown any special hemorrhagic diathesis or tendency.

Of the fever cases, the first was a woman of about forty, married, mother of several children. She had been about two weeks with fever when the hemorrhage began. It commenced unexpectedly and without special premonition. As well as I remember now, there had been no trouble with the bowels in the way of diarrhœa. She had several large discharges of apparently pure blood, much of it clotted, aggregating by estimate, without measuring, three quarts. The complexion was rendered cadaverous, and the prostration very considerable, but after the arrest of the hemorrhage she soon began to revive and gather strength, and the febrile disease was better from the date of the hemorrhage. Her convalescence was not more tedious—was as rapid and satisfactory as that of others that had not been subject to the loss of blood. The treatment consisted in the administration of astringents, such as oak bark, etc., administered both by the mouth and as injections, in large doses and in rapid succession.

The second was a girl, sixteen or seventeen years old. She had been suffering with fever for some time, and was better, had been sitting up, but afterward had been in a relapsed condition for several days when the bleeding commenced. Depletory visitation occurred in the night, and she had, as we may say, two paroxysms of bloody dejections. When I saw her in the morning after the first, I was informed that she had had, during the latter part of the night and early morning, several large, bloody passages, and she showed in her pallid face and by her weak pulse, the effects of the depletion. But at that time the discharge of blood had ceased, and I thought probably might not return. I prescribed ascetate of lead, one or two grains, and opium a fourth of a grain, to be taken at intervals of two or three hours, and inferring that obstruction of the hepatic circulation might have something to do with the hemorrhage, I directed ten grains of blue mass to be taken about noon. At my visit again next morning, I was told the bloody discharge had recurred in the night, commencing this time about nine o'clock, two or three hours earlier than the night before, but had again ceased. I did not see the discharges in this case, but from the account given by the family, the quantity was simply enormous, for one in her condition. The prostration was now extreme and alarming, the pulse barely perceptible, and the palor of the countenance as intense as was possible in a living subject. I administered brandy for present relief, and as it had been paroxysmal, and there seemed a tendency to periodicity, I prescribed quinine fifteen grains, to be taken during the day, and elixir of vitriol ten drops, every hour or two, to prevent recurrence the ensuing night. I remained with her and watched the case that night, but there was no more hemorrhage then nor afterwards. After a few days she began to cönvalesce, though slowly yet surely and steadily to recovery.

The third fever case was that of a man, twenty-eight or thirty years of age. He, too, was in a relapsed condition—had been able to be up and out, but exposed himself too soon. His loss of blood commenced in the early part of the night, and continued into the forenoon of next day. I was absent from home, and did not see him until ten o'clock in the forenoon. He had recently had, as I was informed, the fifteenth discharge when I arrived. It was small, compared with others he had had. It was still in the vessel, and I saw it. It consisted of what appeared to be about a gill of blood, partly clotted. His wife, and a neighbor present, assured me that he had passed in the fifteen discharges, as they believed, at least a gallon of blood. But blood makes a great show, and they may have over-estimated. Before my arrival he had had one dose of precipitate of iron, which I had sent by the messenger, with instructions to give a teaspoonful with a little water every hour, and this fifteenth discharge was the last that occurred at this time after taking the medicine. I repeated it two or three times afterwards, and he complained of uneasiness through the bowels and hips. I gave him a fourth of a grain of morphine to relieve his malaise, quiet his agitation, and procure him sleep. It had the desired effect.

I left him aromatic sulphuric acid, to take in case the hemorrhage should return. Things went on well until late in the ensuing night, when his bowels became uneasy and the discharges commenced again. He took the acid drops, but thought they did not produce so good an effect as the "red powders." So the iron dust was taken freely, and the discharges soon ceased. When I saw him afterwards, I gave him more morphine, and left him some to take in addition to the iron, if needed. There was no further trouble of this kind, and the patient did well.

It may be seen that the iron and morphine were mainly relied on in this case, and that quinine was withheld, that the test of the iron might be more satisfactorily made. I have often used this article for other hemorrhages, and with almost magical effect, but never before for this condition of the bowels. I have great confidence in its usefulness, and may take occasion soon to offer some remarks upon the hæmostatic properties of the precipitated carbonate of iron.

Do not these cases seem to afford grounds for reflection upon the effects of sanguinous depletion in febrile diseases? Here are three cases of fever—the last two not of an active grade, the first was more so, in which not only depletion, but depletion carried to excess, was practiced by natural or diseased process, and which all recovered with but little trouble after the cessation of the hemorrhage. In two of these cases the fever was literally and practically broken, apparently by the hemorrhage; the other had some fever for a few days afterwards. If the abstraction of a little blood from the arm should be regarded with such horror as has become the fashion in these latter days, how is the happy after-result in these cases to be explained?

There are certainly great differences of grade and quality in different cases of fever, both continued and periodic, and the treatment, to be rational, philosophic and successful, must depend, in a great measure, upon these differences, remembering that the tendency in most fevers is to produce local hyperæmias irritations and inflammations, and that an "ounce of preventive" is sometimes "better than a pound of cure;" and, therefore, in such cases as are not marked by prostration and feeble reaction, such restraint should be thrown upon the circulation as will overcome the above-mentioned tendency, or subdue those local conditions if they exist. To this end, revulsions and local and general abstractions and increased activity of secretions, are often beneficial, not directly, but indirectly, by rendering the case more amenable to other treatment. True science knows no prejudice, nor should the medical practitioner know, nor should tolerate, either prejudice or fashion in the treatment of disease and the selection of remedies.

